# Niche Tracking and Rapid Establishment of Distributional Equilibrium in the House Sparrow Show Potential Responsiveness of Species to Climate Change

**DOI:** 10.1371/journal.pone.0042097

**Published:** 2012-07-30

**Authors:** William B. Monahan, Morgan W. Tingley

**Affiliations:** 1 National Park Service, Inventory and Monitoring Division, Fort Collins, Colorado, United States of America; 2 Woodrow Wilson School, Princeton University, Princeton, New Jersey, United States of America; University of Sao Paulo, Brazil

## Abstract

The ability of species to respond to novel future climates is determined in part by their physiological capacity to tolerate climate change and the degree to which they have reached and continue to maintain distributional equilibrium with the environment. While broad-scale correlative climatic measurements of a species’ niche are often described as estimating the fundamental niche, it is unclear how well these occupied portions actually approximate the fundamental niche per se, versus the fundamental niche that exists in environmental space, and what fitness values bounding the niche are necessary to maintain distributional equilibrium. Here, we investigate these questions by comparing physiological and correlative estimates of the thermal niche in the introduced North American house sparrow (*Passer domesticus*). Our results indicate that occupied portions of the fundamental niche derived from temperature correlations closely approximate the centroid of the existing fundamental niche calculated on a fitness threshold of 50% population mortality. Using these niche measures, a 75-year time series analysis (1930–2004) further shows that: (i) existing fundamental and occupied niche centroids did not undergo directional change, (ii) interannual changes in the two niche centroids were correlated, (iii) temperatures in North America moved through niche space in a net centripetal fashion, and consequently, (iv) most areas throughout the range of the house sparrow tracked the existing fundamental niche centroid with respect to at least one temperature gradient. Following introduction to a new continent, the house sparrow rapidly tracked its thermal niche and established continent-wide distributional equilibrium with respect to major temperature gradients. These dynamics were mediated in large part by the species’ broad thermal physiological tolerances, high dispersal potential, competitive advantage in human-dominated landscapes, and climatically induced changes to the realized environmental space. Such insights may be used to conceptualize mechanistic climatic niche models in birds and other taxa.

## Introduction

Species’ distributions are shaped at broad geographic scales by major climatic gradients that interact with the physiology of an organism [1], [2]. When taken in combination, these overlapping climatic limitations on range define the species’ “Grinnellian” fundamental niche [2], [3], or – in revived niche theory parlance – the “scenopoetic” fundamental niche [3], [4]. The range-wide distribution of a species may thus be interpreted as a geographic projection of the scenopoetic fundamental niche (hereafter shortened to “fundamental niche”), formed by the influence of scenopoetic (abiotic) variables such as climate on fitness. Understanding the fundamental niche is highly desired in biology, as accurate descriptions of this niche are critical to understanding, for example, evolutionary processes [5], [6], invasive species risk [7], [8], and climate change impacts [9]–[11]. In practice, however, it is difficult with many organisms to directly measure the fundamental niche, so most studies attempt to reverse engineer it through correlating the observed geographic distribution of a species with abiotic variables to map occupied portions of the fundamental niche (i.e., the “occupied niche”; see Table 1 for comparison of niche definitions derived from Peterson et al. [4]). These purely correlative niche descriptions have received focused scrutiny due to their now widespread use in ecological modeling [12]–[16], and questions have been raised as to what correlative models of the occupied niche actually represent with respect to fitness limitations [17], [18].

**Table 1:** Glossary of niche terminology, adapted from Peterson et al. [4], with reference to the present study; the terms collectively describe three niche classifications that are ordered hierarchically based on their size: fundamental ≥ existing ≥ occupied.

Term	Definition
1. Scenopoetic fundamental niche	The combination of non-consumed, abiotic variables that directly affect an organism physiologically and thereby define the environmental conditions in which the species can persist. Here, equivalent to the abiotic niche, or the Grinnellian niche, and referred to in shorthand as the ‘fundamental niche’.
2. Thermal fundamental niche	A specific type of scenopoetic fundamental niche, defined solely by the physiological tolerance of temperature. Here, referred to simply as the ‘thermal niche.’
3. Existing (thermal) niche	The intersection of the thermal niche with the set of temperatures actually existing at a certain time. Thus, the existing niche is the area within the fundamental niche where a species can potentially occur given the realized environmental space. Equivalent to potential niche, sensu Jackson & Overpeck [23].
4. Physiological niche	A generalized term used here to describe both the fundamental and existing niches when contrasted with the occupied niche.
5. Occupied (thermal) niche	The set of temperatures (or more generally, scenopoetic attributes) associated with the occupied portions of the scenopoetic fundamental niche. The set of conditions included in the occupied niche may be used to calculate summary statistics, as is done in the present study, or as inputs to correlative models (i.e., ecological niche models, sensu Peterson [10], or species distribution models, sensu Guisan & Thuiller [36]). Occupied niches provide the basis for the estimation of a species’ scenopoetic fundamental niche in the absence of physiological data.

The ability of the occupied niche to accurately describe the fundamental niche depends on multiple factors. Chief among these is the extent to which a species has expanded its geographic range to occupy all suitable areas–a condition known as distributional equilibrium [18]–[20]. Distributional equilibrium with respect to an environment is a basic and generally untested assumption of correlative distribution modeling [21], yet is fundamental to predicting species’ responses to future climate change [22]. If a species is in distributional equilibrium, its geographic boundaries may be set by: (i) fitness limits imposed by environmental gradients (e.g., lethal physiological temperatures), or (ii) physical limits of the realized environmental space (e.g., all combinations of temperature that presently exist on the planet). If a species’ distribution is limited by environmental space, then its geographic range will be delineated by the intersection of the fundamental niche with the realized environmental space – that is, the set of all environmental conditions that exist in the geographic domain [23]. This intersection is termed the “existing niche” [4] (Table 1). For correlative models of the occupied niche that are projected forward in time to novel climates [24], [25], a constraint imposed by the assumption of distributional equilibrium is that species lack the physiological capacity to broaden or alter their occupied niche. Correlative models of the occupied niche have had mixed success in predicting changes in distribution over time [26]–[28], suggesting that the assumption of distributional equilibrium may not always be valid. Given these insights, plus evidence from studies suggesting that species are often physiologically capable of persisting in presently unoccupied environments [18], [29], further empirical study is required to better understand the conditions and mechanisms that lead to distributional equilibrium.

Additionally, the evaluation of techniques to quantify characteristics of the occupied niche – whether for descriptive or comparative purposes – has received relatively little attention. Most investigations of the effects of climate change on species’ distributions attempt to model occupied portions of the fundamental niche as a function of a myriad set of scenopoetic variables using one or several correlative statistical methods. While different statistical models are frequently ranked based on the transferability of correlations over space [30], [31] or time [26], [27], broader ecological tests using these models are limited by potential violations of distributional equilibrium and incomplete sampling of environmental space [32], [33]. Furthermore, different correlative modeling methods yield sometimes radically different results (e.g., [30]), thus making simpler measures like the niche center or area – two basic mathematical properties that describe the underlying data used to parameterize all correlative niche models – ideal for testing and evaluating broad-scale environmental drivers of species’ distributions [34], [35]. Such simple and straightforward metrics can be used to ask what physiological niche –fundamental or existing – is best approximated by correlative measures of a species’ occupied niche, how does the occupied niche relate to physiological tolerances, and which, of several, methods of quantitatively describing a niche are preferable.

Given the need for a stronger empirical understanding of the physiological effects of climate on species’ distributions, introduced species offer unique opportunities to further this inquiry as their geographic ranges are often disproportionately limited by scenopoetic factors [8], [36]. In addition, the process of introduction and expansion in contemporary time means that distributional equilibrium may be evaluated without major confounding effects of niche evolution [7]. Thus, introduced species allow us to explore how quickly distributional equilibrium can be reached with respect to physiological tolerances that interact with scenopoetic gradients and – once reached – whether equilibrium is maintained over time as environmental conditions change. In a broader sense, introduced species allow us to quantify and describe one extreme of what is truly a continuum of species’ potential responses to climate change.

Here, we explore these essential questions of geographical ecology by comparing fundamental, existing, and occupied niches in the North American house sparrow (*Passer domesticus*). The house sparrow was first introduced to North America in 1851 [37]–[39], allowing ample time to expand and colonize available climatic space without co-evolved biotic limitations to range. A human commensal, the house sparrow successfully immigrated long distances with people during the late 19^th^ and early 20^th^ centuries [40]; hence, dispersal was not especially limiting. Furthermore, North American house sparrow populations are not sufficiently differentiated genetically from their source European populations to expect niche evolution [41], and previous experimentation on the species’ physiology (e.g., [42]–[44]) provides unusually detailed mechanistic knowledge of its thermal requirements [18].

This unique set of characteristics makes the house sparrow an ideal species for evaluating (i) what physiological niche is best approximated by the occupied niche and (ii) how physical versus physiological limits lead to the establishment and maintenance of distributional equilibrium. We first measure the species’ fundamental niche, as determined by temperature, under different fitness thresholds and using different niche estimators, and then compare the physiologically-defined fundamental and existing niches to the species’ occupied niche. We then consider separately both long-term and interannual temperature changes over a 75-year period (1930–2004) and explore whether, when, and how the species reached distributional equilibrium throughout its introduced North American range.

## Materials and Methods

### Occurrence Data

The two objectives of our study necessarily required us to compile and analyze both contemporary and time series data on the North American distribution of the house sparrow. For the contemporary analyses, we endeavored to illustrate how our methods could be generally applied across taxa and we thus obtained occurrence data from museum collections – a major source of biological data for use in species distribution modeling [45]. For the temporal analyses, we supplemented museum specimens with publically available bird observation data in order to ensure adequate yearly sampling of the species in the contiguous US. We summarize our sampling below, and detail our various data sources, sample sizes, and uses in Table 2.

**Table 2:** Data sources used in the contemporary (C) and temporal (T) niche analyses, including years of coverage and raw sample sizes.

Data Source	Analysis	Years	Sample Size^a^
American Museum of Natural History, New York	C & T	1882–2008	22
North American Bird Banding Program^b^	T	1927–2004	93414
North American Breeding Bird Survey^c^	T	1966–2004	64998
Bishop Museum of Natural History, Honolulu	C & T	1953–2007	27
Burke Museum of Natural History, University of Washington, Seattle	C & T	1886–2005	258
Canadian Museum of Nature, Ottawa	C & T	1879–1985	176
Audubon Christmas Bird Count^d^	T	1902–1998	52448
Cornell University-Museum of Vertebrates, Ithaca	C & T	1900–2004	105
Delaware Museum of Natural History, Wilmington	C & T	1978–2001	184
Denver Museum of Nature and Science	C & T	1897–2007	233
eBird (Cornell Lab of Ornithology)^e^	T	1960–2004	32249
Field Museum of Natural History, Chicago	C & T	1877–2004	407
Great Backyard Bird Count (Cornell Lab of Ornithology)^e^	T	1998–2004	111605
James R. Slater Museum of Natural History, University of Puget Sound, Tacoma	C & T	1900–2000	135
Kansas University Natural History Museum, Lawrence	C & T	2001–2001	1
Los Angeles County Museum of Natural History, Los Angeles	C & T	1890–2004	148
Museum of Southwestern Biology, University of New Mexico, Albuquerque	C & T	1970–1971	9
Museum of Vertebrate Zoology, University of California, Berkeley	C & T	1880–2007	1658
Project FeederWatch (Cornell Lab of Ornithology)^e^	T	1998–2004	456627
Royal Ontario Museum, Toronto	C & T	1867–2004	4297
Santa Barbara Museum of Natural History, Santa Barbara	C & T	1886–2007	63
University of Alaska Museum of the North, Fairbanks	C & T	1987–1993	2
University of California at Los Angeles (Dickey Collection), Los Angeles	C & T	1909–1963	37
University of Colorado Museum of Natural History, Boulder	C & T	1897–1965	13
University of Michigan, Museum of Zoology, Ann Arbor	C & T	1887–2008	105
Yale University Peabody Museum, New Haven	C & T	1878–1987	142

a All sample sizes reflect the total number of georeferenced specimens or observations available at time of data acquisition, including multiple specimens or observations at a given site. Sample sizes are restricted to North America.

b All band and encounter records obtained through written permission from the USGS Bird Banding Lab (http://www.pwrc.usgs.gov/bbl/homepage/datarequest.cfm).

c Obtained from the USGS North American Breeding Bird Survey (http://www.pwrc.usgs.gov/bbs/RawData/Choose-Method.cfm).

d Obtained from the Christmas Bird Count database project (http://infohost.nmt.edu/~shipman/z/cbc/homepage.html).

e All observational data from the Cornell Lab of Ornithology were obtained from the Avian Knowledge Network (http://www.avianknowledge.net/content).

For the contemporary analyses, house sparrow occurrence data were assembled from a complete download (Aug. 18, 2010) of all georeferenced North American specimen records in ORNIS, an online database containing avian specimen data from 42 institutions (http://www.ornisnet.org). After initial removal of specimen records with missing data, 7,824 records from 19 museums were retained, ranging in collection date from 1867 to 2009 (Table 2). For the assessment of the contemporary occupied niche, specimens collected in all years were included because it is well-documented that few populations of the house sparrow in North America have been extirpated once established [38]. Additionally, inclusion of historic occurrence data (pre-1950) was necessary in order to ensure representative sampling of the entire geographic domain. In contrast, the temporal analyses included both georeferenced museum and observational data for the contiguous US, 1930–2004 (Table 2). Temporal niche analyses were restricted to the contiguous US in order to ensure that routine sampling consistently documented the presence of the species each year at the spatial resolution of our analyses (0.5×0.5 decimal degrees, see Climate data, below), and it is for this principal reason that the observation data could not also be included in the contemporary niche analyses, which considered all of North America. While sampling effort (in the form of birds collected or observed) increased between 1930 and 2004, all occurrence data were rendered spatially unique (at the resolution of 0.5×0.5 decimal degrees) in an effort to reduce sampling biases otherwise introduced by targeted sampling in small geographic areas. After aggregation, the contemporary niche analyses were based on a sample size of 1,067 spatially unique localities. Temporal niche analyses were based on 50 randomly selected localities each year, thus ensuring that time series results were not artifacts of increases in sampling intensity across years. We also bootstrapped the localities (generating 100 separate samples, each with 50 localities) to ensure that results were robust to random draw (see Temporal niche analyses, below).

### Physiological Data

Estimates of population mortality with respect to lower and upper ambient temperatures were obtained from previously published experiments involving large numbers of acclimated birds housed under temperature-controlled indoor and outdoor conditions [42]–[44]. These studies were designed to evaluate the relationship between individual energy demand and ambient temperature, including the ambient temperatures that lead to different percentages of population mortality. Because the physiological niche is a fitness gradient (as measured here with respect to temperature), we selected three thermal fitness thresholds for analysis: 100% population mortality (lethal dose [LD]100, minimum  = –35.0°C, maximum  = +47.5°C), 50% population mortality (LD50, minimum  = –23.2°C, maximum  = +42.0°C), and 0% population mortality as defined by the limits of the thermal neutral zone (TNZ, minimum  = +21.5°C, maximum  = +37.0°C). The TNZ is the range of ambient temperatures over which an endotherm can control its internal body temperature without altering its metabolic rate. These temperature limits were applied to the climate data (described below) in order to obtain quantitative estimates of the fundamental and existing niches.

### Climate Data

Gridded climate data were obtained from the Climatic Research Unit [46] for all of North America at a spatial resolution of 0.5×0.5 decimal degrees (0.1°C precision). These data have the highest spatial and temporal resolution combined of any contemporary and historical climate data source covering all of North America. Minimum and maximum mean monthly temperatures were obtained for every year, 1930–2004. From these we computed the minimum temperature of the coldest month and maximum temperature of the warmest month. These temperature gradients were selected because they provide the best match to the physiological data. We computed the monthly winter minima and summer maxima using two approaches. For the contemporary niche analyses, we first averaged separately across years (1950–2000) both minimum and maximum monthly temperature. These years were selected because – based on the weather station data record – they are used by convention to describe ‘contemporary’ climate (e.g., [47]). Then, using these twelve 1950–2000 monthly minimum and maximum temperature averages, we produced two new grids where each cell recorded either minimum temperature of the coldest month or maximum temperature of the warmest month. This approach allowed us to conservatively estimate the two bioclimatic variables based on long-term monthly means. In the temporal niche analyses, we computed minimum temperature of the coldest month and maximum temperature of the warmest month for each year, 1930–2004. Together, these climate data for the geographic domain of North America defined the realized environmental space of our niche analyses.

### Contemporary Niche Analyses

All contemporary niche analyses were based on calculations of (i) the realized environmental space in the period 1950–2000, (ii) the fundamental niche (as defined by physiological limits), (iii) the existing fundamental niche (the intersection of the fundamental niche with the realized environmental space), and (iv) the occupied niche as derived from occurrence data (see Table 1 for niche definitions). These four spaces were evaluated for minimum temperature of the coldest month and maximum temperature of the warmest month, matching precision to the climate data (0.1°C). At this precision, only 12.2% of grid cells in the realized environmental space and 28.5% of grid cells in occupied portions of the fundamental niche correspond to more than one grid cell in geographic space. Given these relatively low percentages, all analyses in climate space were derived from binary, not frequency-based, occurrences.

Niche estimators were quantified using three measures: two area-based measures and one measure of centrality. For the first area estimator, we counted the number of 0.1×0.1°C grid cells in each binary distribution of either the physiological or occupied niche. Cell-based estimates of area are desirable because they are based entirely on observations and thus transparently measure available or occupied climatic niche space. However, they are also sensitive to incomplete species occurrence sampling, so a second area estimator was considered based on the minimum convex polygon. Here, area was calculated from the entire set of grid cells encompassing each minimum convex polygon. This estimator assumes that all temperatures encompassed by the polygons are physiologically suitable for the species, yet some may be unoccupied either due to non-modeled factors or because the temperatures are not presently defined within the realized environmental space. The third estimator was the niche centroid, which we calculated following Tingley et al. [11], using mean grid cell values from binary distributions for the two temperature gradients.

Using these estimators, we calculated the degree of correspondence between the occupied niche and each of its two broader physiological estimates of existing and fundamental niches, evaluated for three fitness thresholds (LD100, LD50, and TNZ). For the niche centroid, correspondence was measured first by calculating the Euclidean distance between the occupied niche centroid and the respective physiological niche centroid. In order to gauge whether a distance was large or small, we also calculated Euclidean distances between the occupied niche centroid and all grid cells encompassed by the focal physiological niche, and using this statistical distribution computed the percentile corresponding to the focal distance. The percentile was subtracted from 100% so that high correspondence was characterized by high percentages and low correspondence was characterized by low percentages. For the two area estimators, we first measured the areas (calculated as the sum of climatic grid cells) encompassed separately by the intersection and the union of the occupied niche and the focal physiological niche. We then divided the area of intersect by the area of union and expressed the resulting ratio as a percentage. As with the centroid, high percentages for the area overlap ratio equate to high correspondence between the two niches, and low percentages equate to low correspondence. Niche estimator correspondence results are only directly comparable within estimator class.

**Figure 1:**
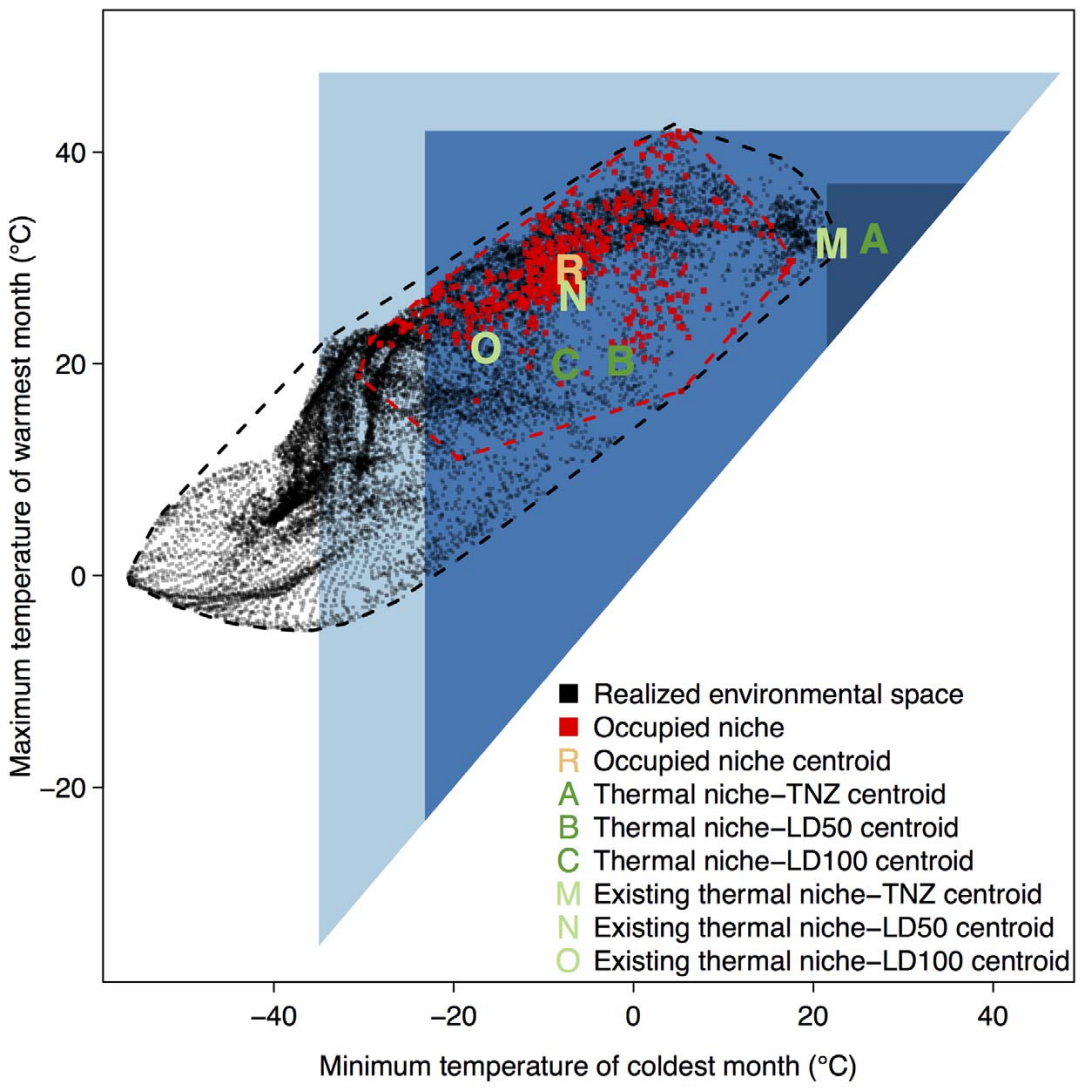
Contemporary niche space for the North American house sparrow, calculated with respect to minimum temperature of the coldest month and maximum temperature of the warmest month. Physiological limits define the boundaries of the triangular fundamental thermal niche for different mortality thresholds (LD100 [light blue], LD50 [medium blue], and TNZ [dark blue]), projected onto the corresponding contemporary realized environmental space for North America (black dots, circumscribed by black minimum convex polygon [MCP]) and the occupied environmental space of the house sparrow (red dots, circumscribed by red MCP). As per definition (Table 1), the intersection of the environmental space with the fundamental niche establishes the existing niche (for a given fitness threshold). Letters identify locations of niche centroids.

**Table 3:** Ability of the occupied niche to describe properties of the physiological niche, comparing across three niche property estimators (centroid, area, MCP), both fundamental and existing niches, and three fitness thresholds (TNZ, LD50, LD100).

Thermal niche type & fitness threshold^a^	Centroid distance				Area overlap^f^			MCP^g^ overlap		
	Min temp^b^	Max temp^c^	Distance^d^	Percentile score^e^	Intersect	Union	Ratio score^h^	Intersect	Union	Ratio score^h^
Occupied niche	−7.12	28.91	0.00	*NA*	4.8	4.8	100.00	815.9	815.9	100.00
Fundamental-TNZ	26.73	31.87	33.98	2.0	0.0	125.7	0.00	0.0	936.1	0.00
Fundamental-LD50	−1.40	20.30	10.34	26.7	4.6	2128.9	0.21	761.1	2180.3	34.91
Fundamental-LD100	−7.50	20.00	8.92	32.8	4.8	3415.2	0.14	815.9	3403.1	23.98
Existing-TNZ	22.06	31.38	29.29	3.1	0.0	5.0	0.00	0.0	817.5	0.00
Existing-LD50	−6.74	26.45	2.49	85.9	4.6	65.4	7.01	760.9	1094.2	69.53
Existing-LD100	−16.39	21.49	11.88	21.5	4.8	112.6	4.23	815.9	1392.2	58.61

a TNZ  =  thermal-neutral zone, LD50 = 50% population mortality, LD100 = 100% population mortality.

b Minimum temperature of the coldest month (°C).

c Maximum temperature of the warmest month (°C).

d Euclidian distance in climate space from the occupied niche centroid to the thermal niche centroid.

e Percentile of occupied centroid distance within distribution of distances from all occupied grid cells to the thermal niche centroid. To ease interpretation in relation to area metrics, percentiles are subtracted from 100%, thus a high percentile score represents a close match between centroids relative to the distribution of all occupied grid cells.

f Area is calculated as the sum of individual 0.1×0.1°C thermal niche grid cells. Areas of occupied cells are compared to areas of the thermal niche. High ratios of overlap indicate higher occurrence within the available environmental space.

g Minimum convex polygon.

h The ratio score is calculated as the area of intersect divided by the area of union, expressed as a percentage.

### Temporal Niche Analyses

Our time series analyses followed from the contemporary niche comparisons, which showed the occupied niche as being best quantified by the centroid and matched by the species’ existing LD50 niche. We thus focused temporal niche analyses on questions of how the existing LD50 niche and the occupied niche in the contiguous US changed over a 75-year period, 1930–2004, as measured by their centroids. While the occupied niche can potentially change over time as a species expands or colonizes new climatic areas, the existing niche can also shift in climate space as geographic areas with climates on the periphery of the fundamental niche either enter into or leave the fundamental niche as the climate changes. Thus, we first evaluated with respect to each temperature gradient whether the existing LD50 and occupied niche centroids changed in a directional fashion through time. This was assessed using simple linear regression by testing for a significant temporal trend in centroid position. The potential effects of serial correlation on these models were explored by calculating the correlation between residuals of linear models and residual lags of one and two years. Only the realized niche centroid as measured by minimum temperature of the coldest month had a significant correlation between residuals and a lag of one year (*P* = 0.02). Since there was no correlation between residuals and a 2-year lag (*P* = 0.88), the linear model for the temporal trend of the realized niche as measured by minimum temperature of the coldest month was also calculated using data from only every other year, and we found our results to be robust. Second, for each temperature gradient, we used Pearson’s correlations to evaluate whether interannual changes in the occupied niche centroid were correlated with interannual changes in the existing LD50 niche centroid.

Beyond the centroid, we also considered more complex temporal changes in both the existing LD50 and occupied niches originating from climatically induced changes in the realized environmental space. We first used simple linear regression to evaluate for each grid cell in each temperature gradient whether climatic changes were directional across years, 1930–2004. We used a statistical cutoff (*P*<0.05) for designation of a linear temporal trend for each grid cell, knowing that true statistical significance for these time-series data would be biased by temporal autocorrelation. Nevertheless, these linear models identified which individual climate grid cells experienced directional changes in each temperature variable, and whether changes were positive or negative over time. To evaluate how these temperature changes corresponded specifically with respect to the physiology of the house sparrow, for each changing grid cell, we subsequently calculated whether the grid cell moved either toward or away from the existing LD50 niche centroid on each temperature gradient. This evaluation allowed us to further determine whether and where niche tracking tended to occur under historical warming and cooling scenarios.

## Results

### Contemporary Niche Dynamics: The Occupied Niche vs. The Fundamental Thermal Niche

The fundamental thermal niche of the house sparrow encompasses many regions of climate space that are outside of the currently observed thermal climate in North America (Fig. 1). Areas within the fundamental niche with no observed realized climate include regions that are warmer than observed climate for minimum temperature of the coldest month, and both warmer and colder than observed climate for maximum temperature of the warmest month. As a result of this mismatch, the occupied niche more closely estimates properties of the existing thermal niche than the fundamental thermal niche, irrespective of fitness threshold and niche estimator (Table 3). The one exception is the thermal niche calculated on a fitness threshold of 100% population mortality (LD100), where the centroid of the occupied niche more closely approximates the fundamental niche than the existing niche (Fig. 1).

**Figure 2:**
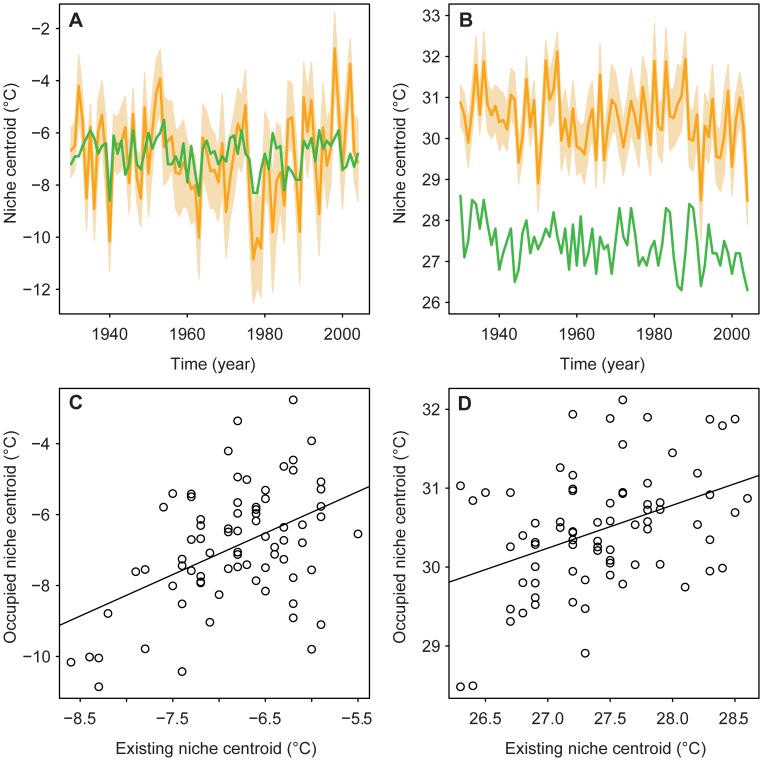
Time series of the occupied and existing LD50 niche centroid locations in climate space: (A) minimum temperature of the coldest month; and (B) maximum temperature of the warmest month. Light orange polygons show the 95% confidence intervals, and dark orange lines the means, of occupied niche centroids evaluated for 100 bootstrap samples (controlling for sample size over time, see Materials and Methods). Green lines show the existing LD50 niche centroids. Plots of the annual location of the occupied niche centroid versus the existing LD50 niche centroid, 1930–2004, for: (C) minimum temperature of the coldest month; and (D) maximum temperature of the warmest month. Solid lines show slope of observed Pearson’s correlations.

For all fitness estimates of both the fundamental and existing niche, the occupied niche centroid showed closer matching to the thermal niche than the two area-based niche measures (Table 3). The first area estimator measured just those grid cells or locations where the species was observed, while the second used these observations to compute a minimum convex polygon. Grid cell-based estimates of correspondence were especially low, owing to the observation that – despite extensive sampling of the thermal niche – many neighboring grid cells in niche space were unoccupied (Fig. 1). However, this finding is sensitive to cell size (here, 0.1×0.1°C), and percentage overlap would be expected to increase with larger grid cells. The minimum convex polygons exhibited higher measures of correspondence (Table 3), but were sensitive to marginal areas of the physiological niches that fell outside the polygon boundaries of the occupied niche (Fig. 1). The niche centroid was largely insensitive to these sampling and edge effects. Compared to the existing LD50 niche centroid, the contemporary occupied niche centroid is nearly aligned with respect to minimum temperature of the coldest month, and it is close, but occurring at slightly warmer temperatures, with respect to maximum temperature of the warmest month (Fig. 1). We thus concluded that – among the measures assessed – the occupied niche most closely approximates the existing LD50 niche centroid.

**Figure 3:**
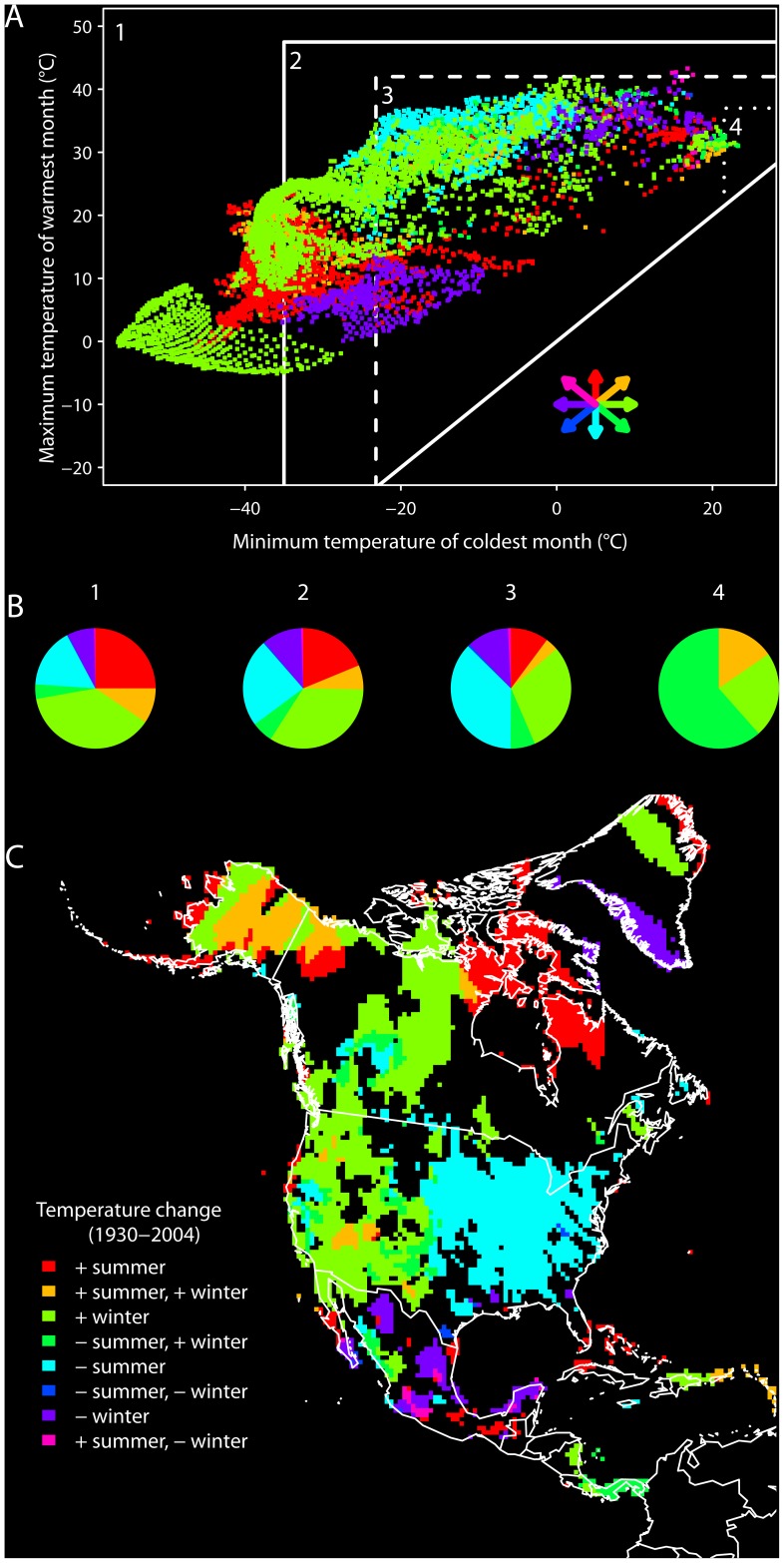
Directional changes in temperature for North America, 1930–2004. (A) The contemporary environmental space showing grid cells that changed in a directional fashion with respect to minimum temperature of the coldest month or maximum temperature of the warmest month (colored grid cells, with directionality symbolized by arrows, matching legend in panel C); grid cells that did not experience directional change are not shown. Labeled regions delineate environmental spaces with respect to different thermal niche fitness thresholds for the house sparrow: (1) the full environmental space; (2) the fundamental LD100 niche; (3) the fundamental LD50 niche; and (4) the fundamental TNZ niche. (B) Pie charts show the proportions of grid cells undergoing each class of directional change within each of the four spaces. (C) Geographic projection of grid cells that experienced directional changes in minimum temperature of the coldest month or maximum temperature of the warmest month. Positive (+)  =  increasing temperature, negative (–)  =  decreasing temperature.

### Temporal Niche Dynamics: How the Occupied Niche Tracked and Filled the Existing Thermal Niche

Having determined how best to infer the house sparrow’s thermal niche, we evaluated how occurrence-based measures of the niche centroid dynamically changed from 1930 to 2004. Our results indicate that the occupied niche of the house sparrow in the contiguous US did not change directionally over time with respect to either minimum temperature of the coldest month (*F*_1,73_<0.01, *P* = 0.99; Fig. 2A) or maximum temperature of the warmest month (*F*_1,73_ = 3.51, *P* = 0.06; Fig. 2B). In other words, there was no systematic temporal trend in the climatic location of the occupied niche centroid toward either warmer or cooler areas. Similarly, the existing LD50 niche over North America did not change directionally with respect to winter minima (*F*_1,73_ = 0.67, *P* = 0.41; Fig. 2A) or summer maxima (*F*_1,73_ = 0.61, *P* = 0.44; Fig. 2B). While centroids for both the occupied and existing niche did not systematically shift in climate space over time, both niche descriptors exhibited interannual differences in temperature. These interannual changes in centroids for the occupied niche and the existing LD50 niche were correlated for both minimum temperature of the coldest month (*t*_73_ = 4.54, *P*<0.0001, *r* = 0.47; Fig. 2C) and maximum temperature of the warmest month (*t*_73_ = 3.79, *P* = 0.0003, *r* = 0.40; Fig. 2D). Combined, these results show that – without systematic or directional movements of the niche centroid over most of the 20^th^ century – interannual changes in the occupied niche have occurred in tandem with interannual changes in the existing LD50 niche, and that the species has essentially been in distributional equilibrium with respect to both temperature variables since the early 20^th^ century.

Beyond the niche centroid, other temporal changes in the occupied and physiological niches are complex, yet not without pattern. Over the same 75-year period, the realized environmental space experienced directional changes in temperature on one or both niche axes (Fig. 3). Of all grid cells in North America, 51.3% exhibited a linear climatic trend on at least one measured temperature gradient. Of these grid cells, there is a clear pattern of warming occurring predominantly in colder environments, and most of this warming can be attributed to increases in minimum temperature of the coldest month (Fig. 3B). Similarly, most cooling has occurred in warmer environments, and most of this cooling can be attributed to decreases in maximum temperature of the warmest month (Fig. 3B). When viewed geographically, most signatures of warming are concentrated in western and northern portions of North America, and most signatures of cooling are restricted to the eastern US and Mexico (Fig. 3C).

**Figure 4:**
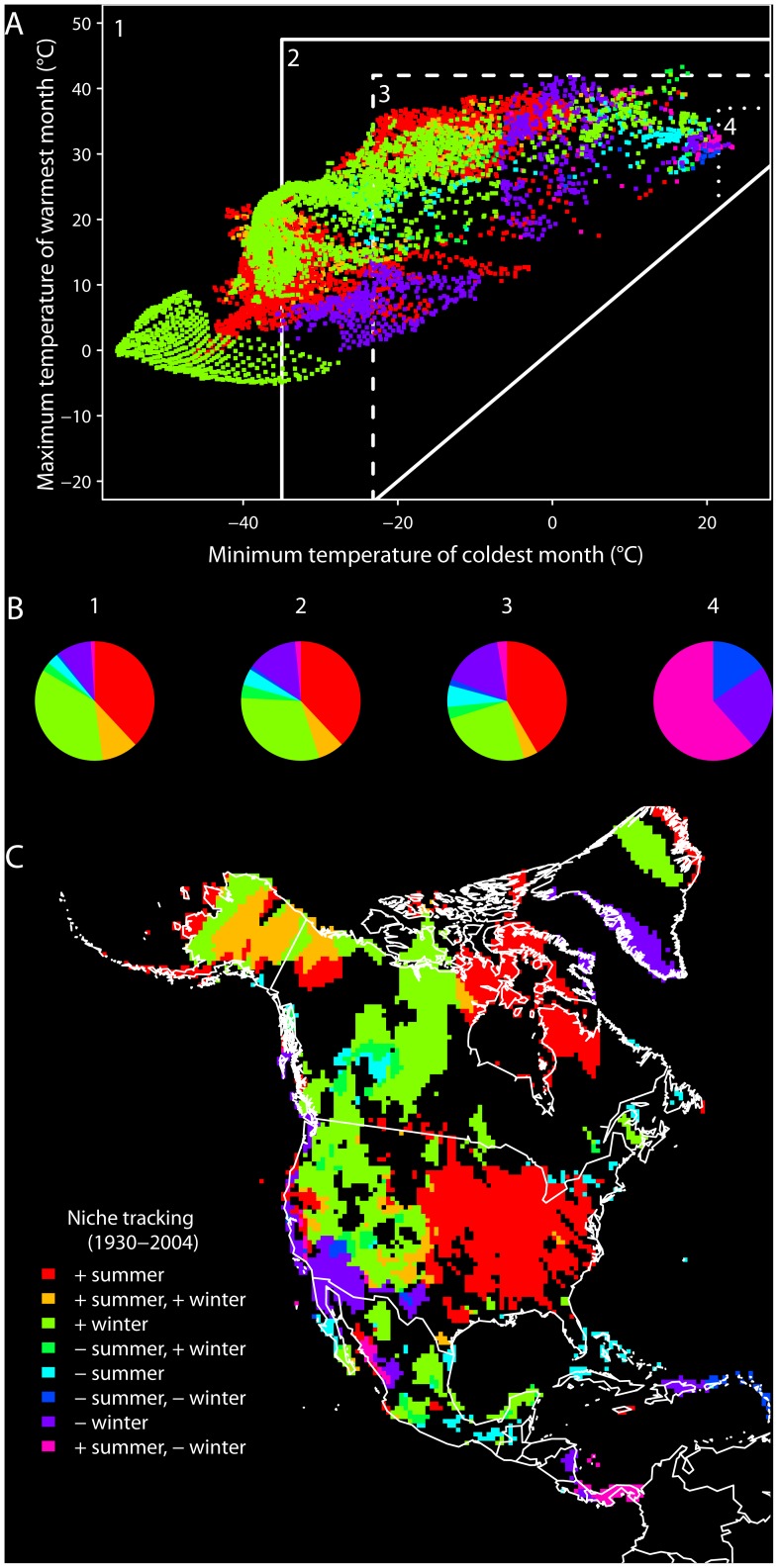
Directional changes in temperature in North America, 1930–2004, with respect to the existing LD50 niche. (A) The contemporary environmental space showing grid cells that moved either toward or away from the existing LD50 niche centroid due to directional changes in minimum temperature of the coldest month or maximum temperature of the warmest month (colored grid cells, legend in panel C); grid cells that did not experience directional change are not shown. Labeled regions delineate four environmental spaces with respect to different thermal niche fitness thresholds for the house sparrow (see Fig. 3). (B) Pie charts show the proportions of grid cells moving either toward or away from the existing LD50 niche centroid within each of the four spaces. (C) Geographic projection of grid cells that moved either toward or away from the existing LD50 niche centroid of the house sparrow. Positive (+)  =  moving toward the niche centroid, negative (–)  =  moving away from the niche centroid.

As a consequence of these temperature changes, the climatic environments of the geographic locations of most North American populations of the house sparrow have shifted to conditions closer to the species’ existing thermal niche centroid (Fig. 4). In total, 87% of all grid cells in the occupied niche that experienced directional temperature change moved toward the existing LD50 niche centroid on one or both temperature axes (Fig. 4B). When considered geographically, signatures of niche tracking dominate the species’ North American range, with the desert southwest as the only large geographic area in the US that has shifted away from the niche centroid (Fig. 4C). Combined, this analysis reveals that throughout much of the 20^th^ century the environmental space for North America and, by extension, the existing niche of the house sparrow, moved centripetally in thermal niche space.

## Discussion

Two fundamental and still largely outstanding questions for the field of geographical ecology are (i) what physiological-mechanistic niche are we approximating when we develop distribution models based on correlations between species’ geographic distributions and major climatic gradients, and (ii) how rapidly can species establish distributional equilibrium when exposed to new environments? Answers to both questions are central to our ability to develop improved forecasts of species’ geographic responses to climate change because they provide insights into both the environmental factors that relate to fitness, which determines the fate of populations, and dispersal, which influences the rate at which new populations may establish and spread across the landscape. In the case of the North American house sparrow, we demonstrate that the species’ occupied thermal niche is most reliably quantified by the centroid and converges on the existing niche centroid calculated on a fitness threshold of 50% population mortality. In addition, we show that this highly vagile and non-native species had established distributional equilibrium by 1930, within 80 years of first introduction, and afterwards tracked its thermal niche on an inter-annual basis. Note that this assessment of distributional equilibrium is fundamentally different from other investigations that instead evaluate in geographic space whether a species fills its predicted distribution based on a correlative model of the occupied niche (e.g., [48], [49]). Our methodology assesses distributional equilibrium as it relates directly to eco-physiological constraints rather than being inferred through hypothesized environmental correlations. Interestingly, previous tests of distributional equilibrium using correlative modeling approaches have found greater divergences from equilibrium [48], [49]. While our findings are limited to a non-indigenous generalist species, they are noteworthy in that they provide novel insights into one rather remarkable extreme of species’ potential responsiveness to climatic temperature change.

The observation that the occupied niche most closely approximates the existing niche rather than the fundamental niche has important consequences for correlative species distribution models projected to other environments. Correlative models are generally trained on present-day climatic associations of species, yet future climate change is anticipated to result in novel no-analog climates [24], [25]. If observed contemporary correlations do not reflect true intrinsic limits of a species in distributional equilibrium, then the resulting models projected to novel environments may under-predict a species’ physiological capacity to track changes in climate. Both the occupied and existing niches are limited in this regard because both are maximally bounded by the realized environmental space. As such, neither provides direct insights into how the species might respond when exposed to novel climates. However, for a highly vagile species like the house sparrow that has essentially filled its existing niche, a plausible hypothesis is that it is poised to colonize other areas of its fundamental niche, provided these new climates become available in North America in the future due to climate change. Such inferences are possible for species that are in distributional equilibrium because their occupied niches are limited by either eco-physiological constraints or by physical constraints of the contemporary realized environmental space.

The significant correlation between annual existing niche centroids – representing physiologically available climates – and annual occupied niche centroids, shows fine-scale inter-annual tracking of the existing LD50 niche by house sparrow populations. This tracking could be enabled by the combination of two processes: (i) directional changes in climate that shift occupied geographical locations to more optimal conditions in niche space, and (ii) fine-scale inter-annual movements of populations responding to climatic variation. Tingley et al. [11] showed that 91% of bird species surveyed in the Sierra Nevada of California tracked their climatic niches over a similar time span on at least one niche axis (summer mean temperature or total precipitation). Our results independently show that the house sparrow tracked its thermal niche throughout most of its North American range, and that the climate of the Sierra Nevada region moved closer to the species’ physiological centroid over the 20^th^ century (Fig. 4C). Evaluating niche dynamics over such a large geographic domain illustrates how both climatic warming and cooling can benefit a species, depending on the position of a population in environmental space relative to its niche centroid. While the centroid did not change directionally through time, many climates in North America did (Fig. 3), and from 1930 to 2004 these directional changes in temperature were centripetal with respect to the house sparrow’s physiologically defined thermal niche (Fig. 4). The net result was a stabilization of the realized environmental space, as manifest through the elimination of certain ‘extreme’ combinations of winter minima and summer maxima that existed historically.

In summary, the introduced house sparrow historically tracked its thermal niche and established distributional equilibrium throughout North America at a rapid and remarkable rate. These dynamics for a highly vagile, non-indigenous generalist species help us to understand one extreme of the continuum of species’ potential responses to ongoing and future climate change. Looking beyond just the house sparrow, the mechanistic analyses presented here are also central to understanding the processes that determine scenopoetic fundamental niches and – by extension – broad-scale geographic distributions in other species. Although in the present study the niche model was parameterized using low-fitness thresholds (i.e., LD50 and LD100), applications of the model in other species need not rely directly on such extreme measures and may instead extrapolate them from experiments that measure individual stress (reduced performance), instead of lethality, at temperatures near the TNZ. Furthermore, because the fundamental niche as considered in a physiological context is a continuously distributed fitness surface that describes the relationship between scenopoetic gradients and other traits that contribute to survival, reproduction, and growth of populations, future applications of the model need not be limited to temperature, or just two niche axes. Armed with new knowledge that could emerge from such applications, we would be in a better position to effectively predict and understand the responses of species to climate and other forms of scenopoetic change [18], [49]. Without this knowledge, our understanding of the past, present, and future distributions of species will be limited to broad-scale contemporary climatic correlations that make implicit assumptions about fitness.
